# Motor and Cognitive Improvements Following Non-Invasive Brain Stimulation with kTMP: A Case Study of Cerebellar Stroke

**DOI:** 10.21203/rs.3.rs-10199168/v1

**Published:** 2026-07-08

**Authors:** Amanda Chirino-Pérez, Christina Merrick, Fredy Miranda-Casasola, Ludovica Labruna, Richard B. Ivry

**Affiliations:** University of California, Berkeley; Magnetic Tides Inc; Universidad Nacional Autónoma de México; Magnetic Tides Inc; University of California, Berkeley

**Keywords:** Cerebellar ataxia, Non-invasive brain stimulation, Postural stability, Cerebellar Cognitive Affective Syndrome, Stroke

## Abstract

Cerebellar ataxia results in debilitating impairments in balance, gait, and cognition, with no established effective treatments. Kilohertz transcranial magnetic perturbation (kTMP) is a novel subthreshold non-invasive brain stimulation (NIBS) method that delivers strong, subthreshold electric fields to the cerebellar cortex without discomfort. We present the case of a 78-year-old patient with cerebellar ataxia following hemorrhagic stroke who underwent amplitude-modulated kTMP (AM-kTMP) targeted to vermal and paravermal cerebellar regions. Active AM-kTMP produced improvements on clinical scales of ataxia alongside enhanced gait stability and cognitive performance, with no device-related adverse reactions, representing a promising NIBS intervention for cerebellar disorders.

## Introduction

The treatment of cerebellar ataxia (CA) presents a formidable clinical challenge. At present, only a single drug has received FDA approval for treating ataxia, with available evidence suggesting modest efficacy that may be offset by the potential for serious side effects [1]. Invasive deep brain stimulation (DBS) has been used in the treatment of CA and other movement disorders associated with cerebellar function, with implants placed either in the cerebellum or thalamic nuclei that receive input from the deep cerebellar nuclei [2]. However, DBS entails significant surgical risk and is likely to be difficult to employ at scale.

The lack of established treatments has led to considerable interest in the potential of non-invasive brain stimulation (NIBS) as a treatment for CA [3, 4]. For example, patients with SCA3 showed a ~ 15% improvement on a standard ataxia rating scale following a 2-week protocol involving repetitive TMS [5]. Improvements have also been observed in studies in which tDCS was used as the NIBS intervention [6, 7]. However, there are also reports of no measurable change following NIBS interventions [3].

There are inherent limitations with current NIBS methods when used to target the cerebellum. Electrical induction methods such as tDCS only induce a small, subthreshold E-field in the brain [8]. This challenge is magnified when targeting deeper structures such as the cerebellum, with average E-field values ranging approximately 0.3–0.4 V/m [9]. While TMS can reach suprathreshold levels, the pulsed nature of the stimulus creates a complex E-field and lacks waveform flexibility. Moreover, TMS can be difficult to tolerate when applied over the cerebellum due to contraction of neck muscles.

With these limitations in mind, we developed a new NIBS method, kilohertz transcranial magnetic perturbation (kTMP) in which a high-current amplifier is used to drive a coil placed on the scalp. Power considerations require that the system operates in the kHz range. By leveraging magnetic induction, kTMP induces significantly stronger subthreshold E-fields than traditional tES; our current kTMP system can reach up to 10 V/m at the cortical surface and 6 V/m at the cerebellar cortex. Amplitude modulation (AM) of the carrier frequency can be tailored to target specific physiological frequencies.

Studies in both human and non-human models have shown that neurons respond to subthreshold kHz stimulation [10]. In studies involving healthy young adults, we have shown that non-modulated kTMP over the primary motor cortex (M1) increases excitability for up to 40 minutes [11]. In pilot work from our group, AM-kTMP in the beta range attenuated motor skill learning in healthy young adults whereas 3 Hz AM-kTMP combined with movement therapy improved upper limb function in chronic stroke patients. Across 433 sessions involving 143 healthy adults and chronic stroke patients, kTMP directed at various brain targets has proven to be safe, highly tolerable, and perceptually indistinguishable from sham stimulation, with no device-related adverse reactions or abnormal muscle activations recorded.

Here, we present the first application of kTMP as an intervention for cerebellar ataxia.

## Case Report

### Patient Presentation and Baseline

**Figure 1:**
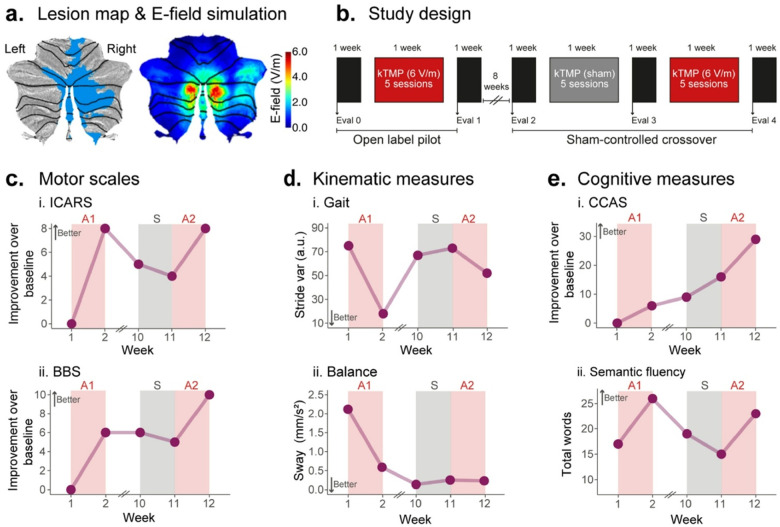
AM-kTMP targeting cerebellar regions produces motor and cognitive improvements in a patient with post-hemorrhagic ataxia. **(A)** Left: Lesion reconstructed from structural MRI and displayed on a cerebellar flat map. Right: Simulated kTMP-induced E-field magnitude and spatial distribution. Coil position induced maximum E-field of ~6 V/m, with distribution across the cerebellar vermis, paravermal regions, and right Crus I/II. **(B)** Study design. Phase 1 consisted of a 1-week open-label pilot. After an eight-week break, Phase 2 comprised a sham-controlled crossover with one week of sham stimulation followed by one week of active AM-kTMP. Evaluations (Eval 0–4) were administered at baseline and three days after the final session of each stimulation week. Red shading = active AM-kTMP; gray shading = sham. **(C)**Clinical motor outcomes. ICARS total score (top) and BBS score (bottom) expressed as improvement over baseline (higher = better). A1 = Active Phase 1; S = Sham; A2 = Active Phase 2. **(D)** Kinematic outcomes. Top: Stride time variability, defined as the coefficient of variation of successive stride intervals, where stride onset is identified from heel-strike troughs in the vertical acceleration signal. Bottom: Mediolateral postural sway measured as trunk acceleration along the x-axis during quiet standing with feet together and eyes open. **(E)** Cognitive outcomes. Top: CCAS Scale total score expressed as improvement over baseline (higher = better). Bottom: Total words produced during the semantic fluency component of the CCAS-Scale assessment

The patient is a 78-year-old female with a history of hypertension and hyperlipidemia who sustained a spontaneous focal cerebellar hemorrhage in 2021 (no evidence of vascular malformation or aneurysm). Structural MRI (Fig. 1A) shows a bilateral, midline lesion centered on the cerebellar vermis, extending into the right cerebellar hemisphere and medial aspects of the right deep cerebellar nuclei. At study onset, three years and two months post-hemorrhage, the patient presented with chronic ataxia predominantly affecting postural control and gait. Baseline score on the International Cooperative Ataxia Rating Scale (ICARS) was 38/100, with the posture and gait disturbance subscale scoring 18/34, while kinetic function, speech, and oculomotor subscales were 17/52, 3/8, and 0/6, respectively. Complementary assessment with the Berg Balance Scale (BBS) yielded a score of 28/56, consistent with moderate balance impairment and elevated fall risk. Cognitive screening using the Cerebellar Cognitive Affective Syndrome (CCAS) Scale showed a baseline score of 84/120. One task fell below cutoff (category switching), meeting criteria for possible CCAS. Verbal fluency scores were also notably reduced relative to test ceiling.

### Study Design

The case study involved two phases (Fig. 1B). The first phase consisted of a 1-week open-label pilot with active AM-kTMP. The 3.5 kHz carrier frequency signal was amplitude modulated at 3 Hz, a value chosen based on prior work showing stimulation in this range promotes inter-regional communication [12]. The coil was positioned 2 cm below and 1 cm to the right of the inion to target midline regions associated with gait and balance [13] and right Crus I/II, regions associated with language function [14]. The current from the amplifier was set to produce an E-field of ~ 6 V/m at the most superficial part of the cerebellar cortex (Fig. 1A).

Stimulation was administered in four 5-min blocks for a total of 20 min. During stimulation, the participant performed a verbal fluency task, naming exemplars of a specified category (e.g., work occupations) or phonemic category (e.g., the letter ‘F’) for 1 min, completing approximately 4 categories per 5-minute stimulation block. Between blocks, the participant performed assisted ambulation and supported single-leg stance.

Assessments were conducted at baseline and on the Monday following each stimulation week (three days after the final session). The assessments included the ICARS, BBS, and CCAS-S, along with kinematic measures of gait and postural stability obtained with a sternum-mounted inertial measurement unit (IMU) that quantifies spatiotemporal gait and balance parameters from trunk accelerations [15]. To minimize practice effects, alternate versions of the CCAS-S were used across assessments, with semantic and phonemic fluency categories unique to each timepoint and distinct from those used during stimulation sessions.

After an eight-week interval, the patient returned for a sham-controlled crossover phase, which began with an assessment of sustained benefits from the first phase. The patient then completed a week of sham stimulation (E-field = 0 V/m), and following a one-week break, a week of active AM-kTMP. The same behavioral protocol was maintained throughout, including fluency tasks during stimulation and gait/balance exercises between blocks.

Following each active kTMP or sham session, the patient rated sensation, annoyance, pain, and muscle twitches on a 0–10 scale (0 = none, 10 = extreme). Ratings were 0 for all measures across all sessions. While the patient was not blinded to treatment allocation during Phase 1, her post-trial report suggested that blinding was preserved during Phase 2.

### Motor and Kinematic Outcomes

The patient exhibited marked motor improvement following active kTMP sessions (Fig. 1C, S1, S2). ICARS total score dropped to 30 after Phase 1, an 8-point improvement over baseline, with the gains confined to the posture and gait disturbance subscale. Improvements were observed in 5 of 7 items, driven primarily by reductions in body sway and improved gait speed. Kinetic, speech, and oculomotor subscales were unchanged. Similarly, the BBS score improved to 34, a 6-point gain following Phase 1.

Kinematic measures corroborated these clinical gains (Fig. 1D). Stride time variability showed a marked reduction following Phase 1, consistent with a more regular and temporally stable gait pattern. Mediolateral postural sway during standing with feet together and eyes open, measured as trunk acceleration along the x-axis, also decreased following active AM-kTMP.

With the exception of stride variability, the gains were generally maintained over the eight-week interval between Phases 1 and 2. Minimal changes were observed on clinical measures following the sham stimulation week in Phase 2. A second week of active AM-kTMP restored ICARS score to post-Phase 1 levels (30/100) and produced further improvement on the BBS, bringing the total improvement to 10 points above baseline (38/56). Stride time variability similarly improved following active but not sham stimulation. Postural sway, already markedly reduced since Phase 1, remained low throughout Phase 2 with no meaningful change following sham or active stimulation.

### Cognitive Outcomes

The patient also exhibited improvement on cognitive measures over the course of the trial (Fig. 1E, S3). CCAS total score improved progressively across all assessment timepoints, increasing by 6 points after Phase 1 (84→90) and reaching a total gain of 29 points by the end of the trial (113/120). By the final assessment, the patient’s diagnostic classification had transitioned from Possible CCAS at baseline to No CCAS. However, as the improvement was also observed following sham stimulation, practice effects cannot be excluded as a contributing factor to the overall score trajectory.

While a case study precludes controlling for category- and letter-specific effects, the semantic fluency data from the CCAS point to a more specific benefit from AM-kTMP. Word output increased substantially following Phase 1 (17 to 26 words) and then declined during the eight-week break, with an additional decline in performance following sham stimulation in Phase 2 that brough performance back to baseline level. The patient exhibited a notable improvement (23 words) following the second active stimulation block. Notably, this improvement was not driven by an increase in the initial semantic burst (0–15 s, baseline = 9 words, active Phase 2 = 9 words), but by better word retrieval during the more demanding, later phases of the 1 min epoch (15–60 s, baseline = 8 words, active Phase 2 = 14 words), suggesting that AM-kTMP facilitated sustained semantic retrieval under increasing cognitive demand.

## Discussion

The findings reported here provide initial evidence that AM-kTMP stimulation over vermal and paravermal regions can produce clinically meaningful improvements in gait and balance, with kinematic measures providing objective corroboration of the clinical gains. The observed 8-point ICARS improvement in postural control and gait after a single week of stimulation in our case study compares favorably with the mean 5-point improvement reported following 15 consecutive days of rTMS in a sham-controlled trial involving a group of 44 SCA3 patients (5). Although the patient was aware of the intervention during the initial phases, the sham-controlled crossover design implemented in Phase 2 provides evidence that the observed benefits were specific to the AM-kTMP stimulation. Specifically, performance remained unchanged following sham stimulation but improved again after the subsequent week of active treatment. Furthermore, the likelihood that these effects reflect spontaneous recovery or non-specific trial-related factors is reduced by the chronic nature of the patient’s condition, as all testing was conducted three years post-hemorrhage.

In terms of cognition, the patient showed continuous improvement over the trial on the CCAS scale. We are hesitant to attribute this to the AM-kTMP stimulation given that the substantial improvement observed between the end of Phase 1 stimulation and start of Phase 2 active stimulation; it may well be that the patient benefitted from repeated testing with the CCAS, even though we used different versions of the test at each assessment. The patient’s performance on the semantic fluency component of the CCAS points to a more specific benefit from AM-kTMP. Here the gains were limited to the assessments following active stimulation. Although there is limited flexibility in coil placement when targeting the cerebellum due to its depth, the induced E-field extended into right Crus I/II, regions associated with fluency impairment (14).

This study has notable limitations, including the single-case design, absence of patient blinding during Phase 1, and an etiology that may differ in neuroplastic potential from progressive degenerative ataxias. However, the results motivate future testing with larger cohorts, particularly hereditary spinocerebellar ataxias, where the therapeutic need is especially pressing. AM-kTMP can deliver strong, subthreshold frequency-specific stimulation to the cerebellum in a tolerable format, making it a promising candidate for a scalable non-invasive therapeutic approach for a condition that has long lacked effective treatments.

## Supplementary Material

This is a list of supplementary files associated with this preprint. Click to download.

- Supplementarymaterial.docx

## Data Availability

The data that support the findings of this study are presented within the article and its supplementary materials. Additional data are not publicly available due to patient privacy considerations but are available from the corresponding author upon reasonable request.
